# CircMYH9/miR-133a-3p/CXCR4 axis: a novel regulatory network in sperm fertilization and embryo development

**DOI:** 10.1186/s43556-024-00236-5

**Published:** 2024-12-13

**Authors:** Qian Sun, Yanyu Li, Wen Yang, Wen Feng, Jiayun Zhou, Yijuan Cao, Bei Zhang, Zuobin Zhu, Conghui Han

**Affiliations:** 1 Suzhou Medical College, Soochow University, Suzhou, 215123 China; 2 Present Address: Department of Gynecology, The First Affiliated Hospital of Kangda College of Nanjing Medical University, Lianyungang, 222061 China; 3 Clinical Medicine Postgraduate Workstation, Soochow University, Xuzhou, 221009 China; 4 Present Address: Department of Gynecology, Xuzhou Central Hospital, No. 199, South Jiefang Road, Quanshan District, Xuzhou, 221009 China; 5 Department of Reproductive Medicine, Xuzhou Central Hospital, Xuzhou, 221009 China; 6 Department of Urology, Xuzhou Central Hospital, Xuzhou, 221009 China; 7 Department of Urology, Xuzhou Clinical School of Xuzhou Medical University, Xuzhou, 221009 China; 8 School of Life Sciences, Jiangsu Normal University, Xuzhou, 221116 China; 9 Department of Urology, Heilongjiang Provincial Hospital, Harbin, 150006 China; 10 Xuzhou Engineering Research Center of Medical Genetics and Transformation, Key Laboratory of Genetic Foundation and Clinical Application, Department of Genetics, Xuzhou Medical University, Xuzhou, 221004 China

**Keywords:** Sperm, CircMYH9, miR-133a-3p, CXCR4, In vitro fertilization rate, Embryo development

## Abstract

This study aimed to investigate the influence of sperm miRNAs on fertilization rates (FR) in in vitro fertilization (IVF) and to explore potential regulatory mechanisms in sperm-mediated fertilization and embryo development. Through high-throughput sequencing, we identified differentially expressed miRNAs in sperm, with miR-133a-3p significantly upregulated in samples associated with low FR and available embryo rate (AER). Key regulatory circRNAs and mRNAs were further identified via the Starbase database, intersected with differentially expressed RNA, and analyzed through GO, KEGG, and PPI analyses. The circMYH9/miR-133a-3p/CXCR4 axis emerged as a critical regulatory network. In vitro assays using the GC-2 spd mouse spermatogenic cell line revealed that miR-133a-3p inhibited cell growth and proliferation while promoting apoptosis. circMYH9, acting as a competing endogenous RNA (ceRNA) for miR-133a-3p, modulated CXCR4 expression, enhancing GC-2 spd cell growth and inhibiting apoptosis through the miR-133a-3p/CXCR4 axis. In vivo experiments using a mouse model confirmed that circMYH9 overexpression increased IVF success rates and promoted embryo development via this axis. Mechanistically, miR-133a-3p suppresses sperm fertilization and embryo development by targeting the circMYH9/miR-133a-3p/CXCR4 axis. These findings suggest that this regulatory network could serve as a novel biomarker for assessing fertilization potential and embryo quality in clinical settings and as a potential therapeutic target to improve IVF outcomes and address infertility. This study provides valuable insights into the molecular mechanisms governing sperm function and early embryonic development.

**Supplementary Information:**

The online version contains supplementary material available at 10.1186/s43556-024-00236-5.

## Introduction

In vitro fertilization (IVF) is a widely used technology in reproductive medicine that has helped many couples struggling with infertility achieve their dream of parenthood [1, 2]. The core of IVF involves combining sperm and egg in a laboratory setting to form a fertilized embryo, which is then implanted into the uterus. Despite the success of IVF, its fertilization rate (FR) and embryo development success rate remain influenced by various factors [3]. In recent years, researchers have increasingly focused on small RNAs in sperm, especially microRNAs (miRNAs), which may play key roles in regulating sperm function and enhancing IVF success rates [4].

MiRNAs are a class of non-coding RNA molecules that regulate gene expression post-transcriptionally by targeting specific mRNAs [5], affecting various biological processes such as cell proliferation, differentiation, and apoptosis [6]. Studies have shown that miRNAs play essential roles in sperm maturation, motility, and fertilization [7]. Additionally, changes in the expression of certain miRNAs have been closely associated with male infertility [8], suggesting that these miRNAs are crucial to fertility. This has prompted scientists to explore their potential regulatory roles in IVF, with the aim of developing new strategies to improve fertilization and embryo development success rates [9].

Among these, miR-133a-3p is known for its regulatory role across various biological processes and systems, including muscle development, cell proliferation and apoptosis, cancer, cardiovascular function, and the reproductive system [10, 11]. Previous studies have indicated that miR-133a-3p is highly expressed in sperm with low FR, suggesting it may have an inhibitory effect on IVF [12]. However, the regulatory mechanisms of miR-133a-3p in fertilization and embryo development remain unclear and warrant further investigation. In addition, circMYH9, a circular RNA (circRNA) formed from the MYH9 gene via back-splicing, possesses high stability and specificity due to its circular structure and plays significant roles in cell proliferation, migration, and apoptosis [13, 14]. Concurrently, CXCR4, a chemokine receptor, is involved in regulating cell apoptosis and migration and is considered a potential biomarker for predicting embryo implantation success in assisted reproductive technology [15]. Based on this background, we hypothesized that circMYH9 and CXCR4 might be key regulatory factors associated with miR-133a-3p.

In summary, this study utilized high-throughput sequencing and bioinformatics analysis to construct the circMYH9/miR-133a-3p/CXCR4 regulatory network, validating its roles in sperm growth, proliferation, and apoptosis, and further confirming its regulatory influence on IVF FR and embryo development. These findings not only deepen our understanding of the role of sperm miRNAs in the IVF process but also provide new potential targets for improving IVF success rates in future clinical applications. This research offers novel therapeutic approaches for treating infertility and establishes an important theoretical foundation for future studies.

## Results

### Key role of miR-133a-3p in sperm-mediated IVF and embryo development

**Fig. 1:**
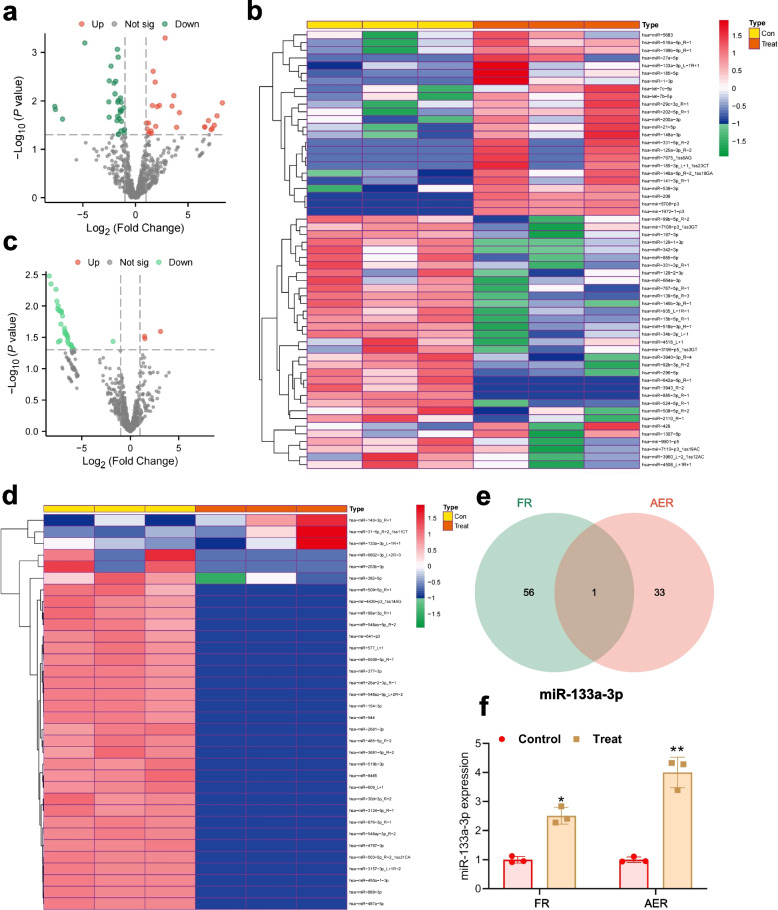
Key miRNAs identified through bioinformatics analysis in the IVF process. Note: **a**-**b** Volcano plots and heatmaps showing differentially expressed miRNAs grouped according to FR. Red represents upregulated genes; blue represents downregulated genes. The control group consists of 3 samples, and the treatment group consists of 3. **c**-**d** Volcano plots and heatmaps showing differentially expressed miRNAs grouped according to AER. Red represents upregulated genes; blue represents downregulated genes. The control group consists of 3 samples, and the treatment group consists of 3. **e** Venn diagram representing the intersection of differentially expressed miRNAs: miR-133a-3p. **f** Upregulation of miR-133a-3p expression in the FR and AER experimental groups compared to the control group (*P < 0.05, **P < 0.01)

In order to investigate the impact of miRNA and its regulatory network on the IVF process, we conducted a differential analysis of miRNA based on sequenced clinical samples, identifying differentially expressed miRNAs through high-throughput sequencing analysis and determining key regulatory miRNAs. Differential analysis of miRNA was performed based on FR grouping, resulting in 26 significantly upregulated genes and 31 significantly downregulated genes (Fig. 1a-b). To further pinpoint key miRNA in sperm IVF, we conducted miRNA differential analysis based on the clinical trait of AER, identifying 3 significantly upregulated genes and 31 significantly downregulated genes (Fig. 1c-d). Intersection of the above differential miRNAs revealed key miRNAs, including miR-133a-3p (Fig. 1e). Subsequently, we compared the miR-133a-3p expression statuses in the low FR group and the low AER group from the sequencing data, observing an upregulation in both groups (Fig. 1f). Therefore, we hypothesize that miR-133a-3p may impact IVF and embryo development by influencing sperm growth, apoptosis, and other physiological processes.

### miR-133a-3p modulates growth, proliferation, and apoptosis in GC-2spd germ cells

**Fig. 2:**
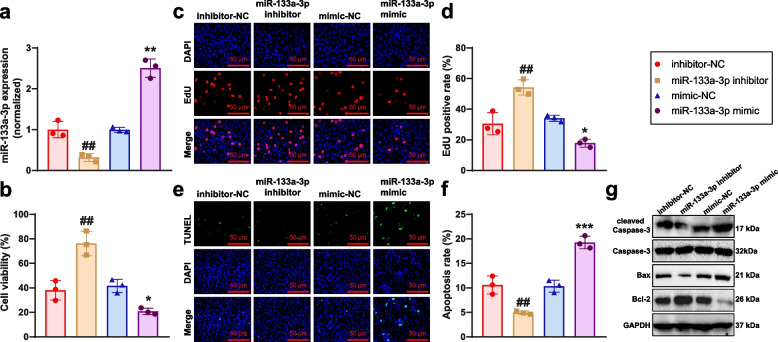
Biological effects of miR-133a-3p on GC-2spd cells. Note: **a** RT-qPCR analysis of miR-133a-3p expression levels in each group. **b** MTT assay to measure cell viability in each group. **c** EdU experiment to assess cell proliferation capacity (scale bar: 50 μm). **d** Statistical analysis of EdU experiment results. **e** TUNEL assay to determine apoptosis rate in each group (Scale bar = 50 μm). **f** Statistical analysis of TUNEL experiment results. **g** Western blot analysis of Caspase-3, cleaved Caspase-3, Bax, and Bcl-2 protein expression levels in each group. Comparisons were made to the mimic-NC group (*P < 0.05, **P < 0.01, ***P < 0.001, ##*P* < 0.01). Cell experiments were performed in triplicate

The research demonstrates that miR-133a-3p can impact cell survival [16]. We investigated the influence of miR-133a-3p on GC-2spd spermatogonia cells and validated its effects through overexpression and knockdown experiments. RT-qPCR confirmed significant upregulation of miR-133a-3p in the mimic group and downregulation in the inhibitor group compared to their respective controls (Fig. 2a). As a result, we successfully established GC-2spd cell models with miR-133a-3p overexpression or silencing for further experiments.

In a study of miR-133a-3p effects on GC-2spd cells, MTT assay showed reduced cell viability, EdU experiment indicated decreased cell proliferation, and TUNEL staining demonstrated elevated apoptosis in the miR-133a-3p mimic group compared to mimic-NC (Fig. 2b-g); whereas compared to the inhibitor-NC group, the miR-133a-3p inhibitor group showed notably strengthened cell viability and proliferation, with reduced levels of cell apoptosis (Fig. 2b-g). It is indicated by the results that miR-133a-3p can impede the proliferation and growth of GC-2spd cells and stimulate cell apoptosis.

### Construction and analysis of the ceRNA network: implicating circMYH9, miR-133a-3p, and CXCR4 in IVF and embryo development

**Fig. 3:**
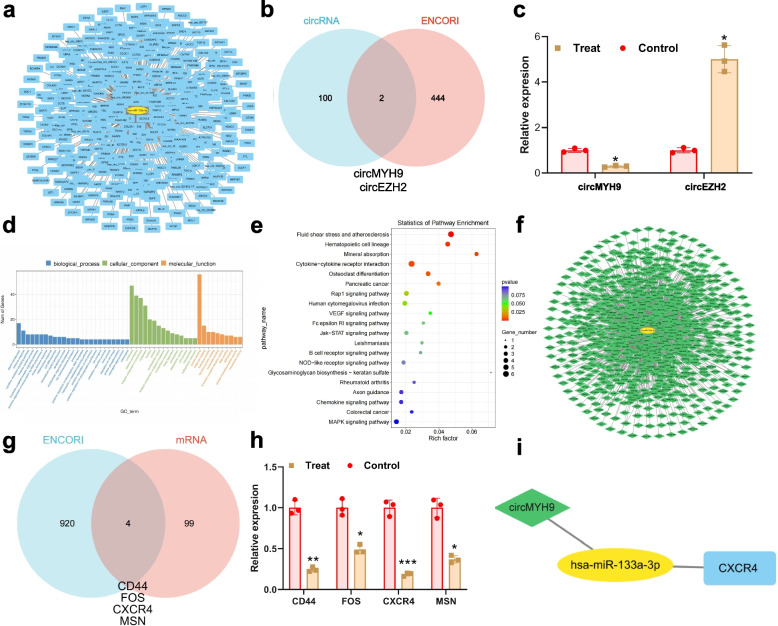
Identification of Key mRNA and circRNA in the IVF Process through Bioinformatics Screening. Note: **a** miRNA-circRNA regulatory network, where yellow represents miRNA and green represents circRNA. **b** Venn diagram representing the intersection of predicted miRNA target circRNAs from the Starbase database and differentially expressed circRNAs. **c** Relative expression levels of circRNA circMYH9 and EZH2 in the experimental and control groups (n = 2). *P < 0.05, **P < 0.01, ***P < 0.001 compared to the control group. **d** Enrichment analysis of differentially expressed mRNAs using GO. **e** Enrichment analysis of differentially expressed mRNAs using KEGG. **f** miRNA-mRNA regulatory network, where yellow represents miRNA and blue represents mRNA. **g** Venn diagram representing the intersection of predicted miRNA target mRNAs from the Starbase database and differentially expressed mRNAs analyzed by PPI. **h** Relative mRNA CD44, FOS, CXCR4, and MSN expression levels in the experimental and control groups (*n* = 3). **i** circMYH9/miR-133a-3p/CXCR4 regulatory network, where yellow represents miRNA, blue represents mRNA, and green represents circRNA

To construct a comprehensive ceRNA network, differential analysis was performed on circRNAs and mRNAs. The results revealed 57 significantly upregulated genes and 82 significantly downregulated genes in circRNA analysis (Figure S1a-c), 167 significantly upregulated genes and 502 significantly downregulated genes in mRNA analysis (Figure S1b-d). Utilizing the identified disease-associated miRNA, miR-133a-3p, predictions of circRNAs interacting with it were made using the StarBase database (Fig. 3a). The intersection of these predictions with differentially expressed circRNAs led to the identification of key circRNAs: circMYH9 and EZH2, where circMYH9 was downregulated and EZH2 was upregulated (Fig. 3b-c). This suggests circMYH9 as a potential ceRNA for miR-133a-3p.

More in-depth analysis was executed by carrying out GO and KEGG enrichment analysis on differentially expressed mRNAs. The GO analysis indicated that the BP these mRNAs may be involved in include signal transduction, immune response, and cytokine-mediated signaling pathways. Their potential roles encompass cellular components such as the cell membrane, membrane, and membrane components, and their molecular functions could include protein binding, metal ion binding, and hydrolase activity (Fig. 3d). KEGG analysis revealed potential regulatory pathways that the target genes may participate in, such as Fluid shear stress and atherosclerosis, Hematopoietic cell lineage, and Mineral absorption (Fig. 3e). Additionally, a PPI network analysis using the STRING database on differentially expressed mRNAs unveiled core regulatory genes (Figure S2).

Furthermore, predictions of miRNAs binding to mRNAs were made using StarBase (Fig. 3f), and an intersection was taken with the core regulatory genes identified in the PPI analysis, resulting in key downregulated mRNAs: CD44, FOS, CXCR4, and MSN (Fig. 3g-h). Based on the differential genes and enriched gene analysis from the databases, it is hypothesized that miR-133a-3p may regulate sperm IVF and embryo development processes through the circMYH9/miR-133a-3p/CXCR4 network (Fig. 3i).

### Validating the targeted association: miR-133a-3p Binds with circMYH9 and CXCR4, establishing circMYH9 as a ceRNA influencing CXCR4 function

**Fig. 4:**
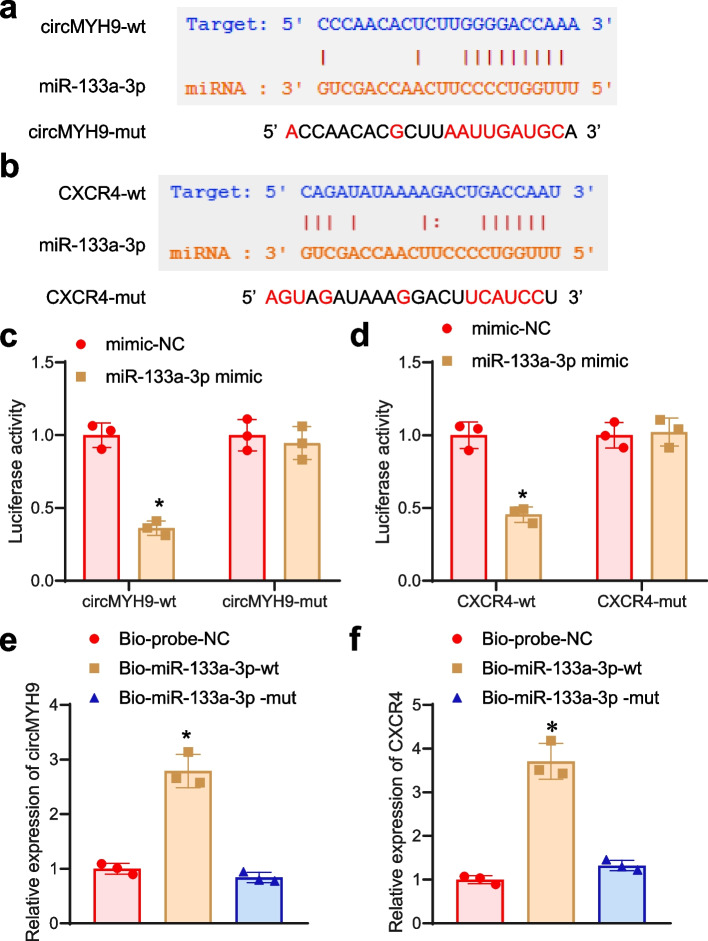
Validation of the interaction among circMYH9/miR-133a-3p/CXCR4. Note: **a** Prediction of the binding site between miR-133a-3p and circMYH9(wt), where circMYH9(mut) denotes the mutated sequence. **b** Prediction of the binding site between miR-133a-3p and CXCR4(wt), where CXCR4(mut) denotes the mutated sequence. **c** Dual-luciferase reporter assay to detect the targeting relationship between miR-133a-3p and circMYH9 (experiment repeated three times, *P < 0.05 compared to the mimic-NC group). **d** Dual-luciferase reporter assay to detect the targeting relationship between miR-133a-3p and CXCR4 (experiment repeated three times, *P < 0.05 compared to the mimic-NC group). **e** RNA pull-down assay to investigate the interaction between miR-133a-3p and circMYH9 (experiment repeated three times, *P < 0.05 compared to Bioprobe NC or Bio-miR-133a-3p-mut). **f** RNA pull-down assay to investigate the interaction between miR-133a-3p and CXCR4 (experiment repeated three times, *P < 0.05 compared to Bioprobe NC or Bio-miR-133a-3p-mut)

Based on the predictions from Starbase databases, we identified the binding sites of miR-133a-3p with circMYH9/CXCR4 and designed mutant sequences for circMYH9 and CXCR4 (Fig. 4a-b). Initially, we executed experiments using the dual-luciferase reporter gene system. The results revealed a marked decrease in luciferase activity in the miR-133a-3p mimic group, contrasting with the mimic-NC group in circMYH9-wt (Fig. 4c), indicating the putative interaction of miR-133a-3p with circMYH9. Similarly, in CXCR4-wt, the luciferase activity in the miR-133a-3p mimic group significantly decreased when juxtaposed with the mimic-NC group (Fig. 4d), suggesting the interaction between miR-133a-3p and CXCR4. Furthermore, it was evidenced by the results of the RNA pull-down study that the enrichment levels of circMYH9 and CXCR4 exhibited a notable escalation in the Bio-miR-133a-3p-wt group compared to the Bio-probe-NC and Bio-miR-133a-3p-mut groups (Fig. 4e-f), validating the connection between miR-133a-3p and circMYH9/CXCR4. To sum up, based on these discoveries, we have identified that miR-133a-3p indeed targets circMYH9/CXCR4, where circMYH9 serves as a competitive endogenous RNA for miR-133a-3p, thereby influencing the functionality of CXCR4.

### miR-133a-3p modulates growth, proliferation, and apoptosis in GC-2spd Cells via regulation of CXCR4 expression

**Fig. 5:**
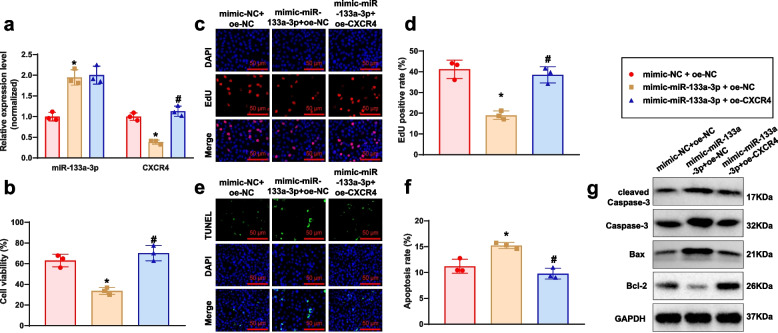
The Impact of miR-133a-3p regulation on CXCR4 in GC-2spd cell biological functions. Note: **a** Expression levels of miR-133a-3p and CXCR4 were measured using RT-qPCR; **b** Cell viability in each group was assessed using the MTT assay; **c** Proliferation ability of cells in each group was evaluated through the EdU assay (scale bar: 50 μm); **d** Statistical analysis of the EdU assay results; **e** Apoptosis rates of cells in each group were determined using the TUNEL assay (scale bar: 50 μm); **f** Statistical analysis of the TUNEL assay results; **g** Protein expression levels of Caspase-3, cleaved Caspase-3, Bax, and Bcl-2 in each group were detected by Western Blot; * denotes a difference compared to the mimic-NC + oe-NC group (*P* < 0.05), # denotes a difference compared to the mimic-miR-133a-3p + oe-NC group (*P* < 0.05), all cell experiments were performed in triplicate

We overexpressed miR-133a-3p alone or with CXCR4 in GC-2spd cells to study their effects. Results showed elevated miR-133a-3p and decreased CXCR4 in mimic-miR-133a-3p + oe-NC group. In mimic-miR-133a-3p + oe-CXCR4 group, miR-133a-3p levels were unchanged, but CXCR4 expression recovered (Fig. 5a). Subsequent MTT assays, EdU experiments, TUNEL staining, and Western blot analysis revealed that the mimic-miR-133a-3p + oe-NC group displayed decreased cellular viability, weakened cell proliferation, and increased apoptosis when contrasted with the mimic-NC + oe-NC group. However, these effects were reversed upon the overexpression of CXCR4 on this basis (Fig. 5b-g).

These outcomes illustrate that miR-133a-3p suppresses the growth and proliferation of GC-2spd cells and stimulates cell apoptosis by regulating CXCR4 expression.

### MYH9 enhances GC-2spd cell proliferation and inhibits apoptosis by modulating the miR-133a-3p/CXCR4 regulatory axis

**Fig. 6:**
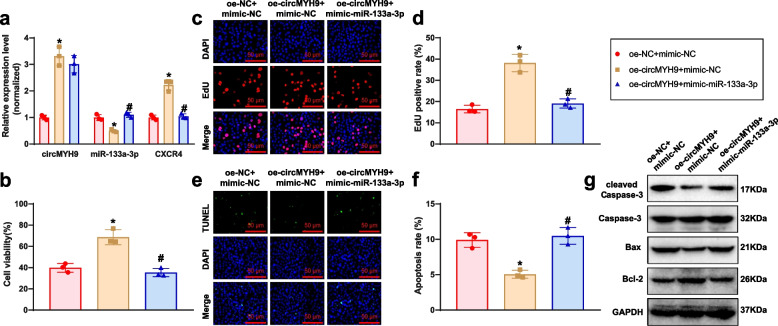
The Impact of circMYH9/miR-133a-3p/CXCR4 Axis on GC-2spd cell biological functions. Note: **a** Expression levels of circMYH9, miR-133a-3p, and CXCR4 were measured using RT-qPCR; **b** Cell viability in each group was assessed using the MTT assay; **c** Proliferation ability of cells in each group was evaluated through the EdU assay (scale bar: 50 μm); **d** Statistical analysis of the EdU assay results; **e** Apoptosis rates of cells in each group were determined using the TUNEL assay (scale bar: 50 μm); **f** Statistical analysis of the TUNEL assay results; **g** Protein expression levels of Caspase-3, cleaved Caspase-3, Bax, and Bcl-2 in each group were detected by Western Blot; * denotes a difference compared to the oe-NC + mimic-NC group (*P* < 0.05), # denotes a difference compared to the oe-MYH9 + mimic-NC group (*P* < 0.05), all cell experiments were performed in triplicate

To investigate the effects of circMYH9 on GC-2spd cells through the miR-133a-3p/CXCR4 axis, circMYH9 and miR-133a-3p were overexpressed using lentivirus transfection. RT-qPCR revealed that in the oe-MYH9 + mimic-NC group, circMYH9 and CXCR4 expression increased while miR-133a-3p expression decreased. Overexpression of miR-133a-3p counteracted the circMYH9-induced suppression of miR-133a-3p, resulting in reduced CXCR4 expression (Fig. 6a). The outcomes indicated that the oe-MYH9 + mimic-NC group demonstrated higher cell viability and proliferation capacity than the oe-NC + mimic-NC group. Additionally, this group exhibited a decrease in the level of cellular apoptosis. The cell viability and proliferation ability of the oe-MYH9 + mimic-miR-133a-3p group exhibited a reduction in contrast to the oe-MYH9 + mimic-NC group. Additionally, elevated levels of cellular apoptosis were detected in the oe-MYH9 + mimic-miR-133a-3p group (Fig. 6b-g).

**Fig. 7:**
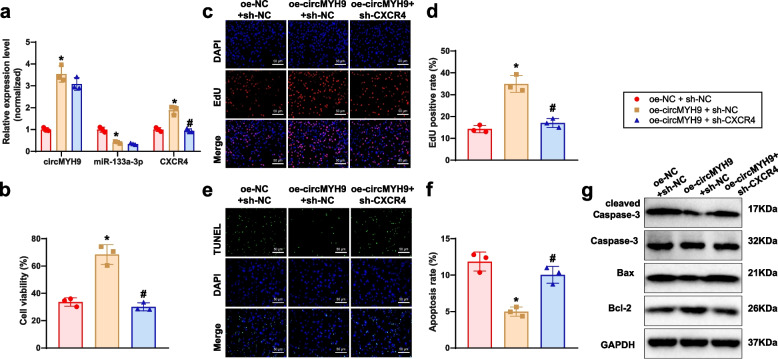
Effects of the circMYH9/miR-133a-3p/CXCR4 axis on the biological functions of GC-2spd cells. Note: **a** RT-qPCR analysis of circMYH9, miR-133a-3p, and CXCR4 expression levels; **b** MTT assay to assess cell viability in each group; **c** EdU assay to evaluate cell proliferation in each group (scale bar: 50 μm); **d** Statistical analysis of the EdU assay results; **e** TUNEL assay to detect apoptosis rates in each group (scale bar: 50 μm); **f** Statistical analysis of TUNEL assay results; **g** Western blot analysis of Caspase-3, cleaved Caspase-3, Bax, and Bcl-2 protein expression levels in each group. * indicates *P* < 0.05 compared to the oe-NC + sh-NC group, # indicates *P* < 0.05 compared to the oe-circMYH9 + sh-NC group. All cell experiments were repeated three times

We overexpressed circMYH9 or co-overexpressed circMYH9 and silenced CXCR4 in GC-2spd cells. Results showed circMYH9 upregulation, miR-133a-3p downregulation, and CXCR4 elevation in oe-circMYH9 + sh-NC group. Silencing CXCR4 reversed circMYH9's effect on CXCR4 without affecting miR-133a-3p expression (Fig. 7a). The MTT, EdU, TUNEL, and Western blot results showed that compared to the oe-NC + sh-NC group, the oe-circMYH9 + sh-NC group exhibited significantly enhanced cell viability and proliferation, with reduced apoptosis levels. In contrast, the oe-circMYH9 + sh-CXCR4 group showed significantly reduced cell viability and proliferation, with increased apoptosis levels (Fig. 7b-g).

The outcomes described above imply that circMYH9 is capable of boosting the growth and proliferation of GC-2spd cells while also preventing cell apoptosis through the regulation of the miR-133a-3p/CXCR4 signaling pathway.

### MYH9 overexpression enhances IVF and early embryo development via the miR-133a-3p/CXCR4 axis in a mouse model

**Fig. 8:**
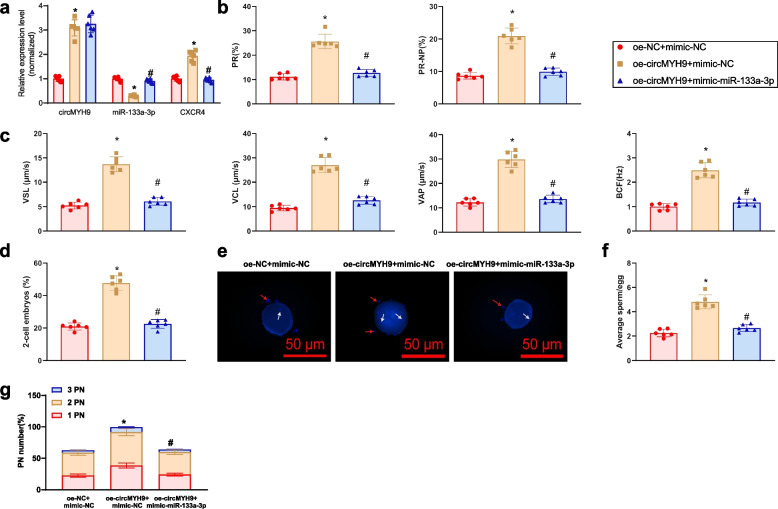
The Impact of circMYH9/miR-133a-3p/CXCR4 Axis on Mouse FR and Early embryo development. Note: **a** RT-qPCR analysis of circMYH9, miR-133a-3p, and CXCR4 expression levels; **b** Analysis of sperm motility parameters in each group using the CASA system, including progressive motility rate (PR) and total motility rate (PR + NP), where NP represents the non-progressive motility rate; **c** Analysis of sperm motility parameters in each group using the CASA system, including curvilinear velocity (VCL), straight-line velocity (VSL), average path velocity (VAP), and beat cross frequency (BCF); **d** Statistical data on the proportion of 2-cell stage embryos after sperm-oocyte binding in each group; **e** Observation of sperm attachment (white arrows) 4 h after fertilization using DAPI staining, with red arrows indicating cumulus cells (scale bar: 50 μm); **f** Statistical chart of the number of sperm attached to each fertilized egg; **g** Observation of the percentage of embryos with 1, 2, or 3 pronuclei (PN) during early fertilization using TLI. * denotes a difference compared to the oe-NC + mimic-NC group (*P* < 0.05), # denotes a difference compared to the oe-MYH9 + mimic-NC group (*P* < 0.05), each group consisted of 6 mice

To evaluate the impact of circMYH9/miR-133a-3p/CXCR4 on embryo development, lentiviral transfections overexpressing circMYH9 and co-overexpressing circMYH9 and miR-133a-3p were constructed in a mouse model. RT-qPCR results showed increased circMYH9 and CXCR4 expression and decreased miR-133a-3p in the oe-MYH9 + mimic-NC group, compared to the oe-NC + mimic-NC group. However, miR-133a-3p expression was restored, and CXCR4 expression was decreased in the oe-MYH9 + mimic-miR-133a-3p group (Fig. 8a). The results affirm the efficient creation of a mouse model that could be utilised for future research.

Sperm motility analysis revealed significant increases in PR, PR + NP, VCL, VSL, VAP, and BCF in the oe-circMYH9 + mimic-NC group compared to the control, but these parameters were reduced in the oe-circMYH9 + mimic-miR-133a-3p group (Fig. 8b-c).

Co-culture experiments showed increased 2-cell embryos in the oe-MYH9 + mimic-NC group and a decrease in the oe-MYH9 + mimic-miR-133a-3p group (Fig. 8d).

Sperm binding to the zona pellucida and the development ratio of fertilized embryos were higher in the oe-MYH9 + mimic-NC group compared to the control, while both were reduced in the oe-MYH9 + mimic-miR-133a-3p group (Fig. 8e-g). The results suggest that circMYH9 overexpression promotes sperm IVF rates and early embryo development, potentially through the miR-133a-3p/CXCR4 axis.

## Discussion

Research indicates that miRNAs are vital in reproduction, such as in regulating sperm formation, maturation, and function [11]. Studies have also found that miR-133a-3p is significantly upregulated in low FR and low AER groups [11]. However, the operational mechanism of miR-133a-3p in IVF remains unclear [17]. The utilization of high-throughput sequencing techniques was pivotal in the detection of varied expression levels of miRNAs linked to IVF FR and the developmental stages of embryos, particularly miR-133a-3p, further validating the key role of miRNAs in reproduction. This aligns with existing research and deepens our understanding of specific miRNAs in the IVF process. Our study fills this gap by clarifying the regulatory roles in IVF and embryo development.

Existing studies often utilize GO and KEGG functional and pathway enrichment analysis to reveal the biological functions and involved signaling pathways of differentially expressed mRNAs [18–20]. Similar analyses in our study identified key mRNAs related to IVF FR and embryo development. Together with miRNA and circRNA regulatory networks, this further unveils the specific regulatory mechanisms of sperm miRNAs in reproduction. This consistency with existing research methods and results enhances the scientific rigor and credibility of our study.

In recent years, the role of circRNAs as ceRNAs in modulating gene expression has garnered attention. Studies indicate that the regulation of miRNA target genes can be modulated by circRNAs through the sequestration of miRNAs [13]. While studies on sperm IVF FR exist, few have addressed early embryo development [21]. By predicting miRNA-binding circRNAs and combining them with differentially expressed circRNAs, we constructed the circMYH9/miR-133a-3p/CXCR4 regulatory network, validating the regulatory role of circRNAs in IVF. This finding corresponds with existing research, further confirming the importance of circRNAs in reproduction.

Our in vitro cellular experiments validated the specific roles of miR-133a-3p, circMYH9, and CXCR4 in sperm growth, proliferation, and apoptosis, with in vivo animal experiments further confirming their regulatory roles in IVF FR and embryo development. This experimental design and validation method align highly with existing research, ensuring the reliability and repeatability of our results. Existing research has shown that sperm quality and function directly impact IVF success rates [22]. Our study reveals how sperm miRNAs and their regulatory networks affect IVF FR and embryo development, offering new potential targets to improve IVF success rates. This finding aligns with current research objectives, further enriching the studies aimed at enhancing IVF success rates. This provides a new perspective in the field of reproduction, allowing researchers to delve deeper into the role of miRNAs in sperm fertilization and embryo development.

The circMYH9/miR-133a-3p/CXCR4 axis has been identified as a crucial regulatory network. Research on this network not only provides a deeper understanding of gene regulation mechanisms but also offers new strategies and targets for treating infertility and embryo development disorders. This study demonstrates that miR-133a-3p, via the circMYH9/miR-133a-3p/CXCR4 axis, inhibits sperm fertilization and embryo development in vitro. This provides clinicians with new biomarkers that may hold significant value in assessing fertilization potential and embryo quality. Moreover, modulation of this regulatory axis could present new treatment opportunities for infertility patients.

**Fig. 9:**
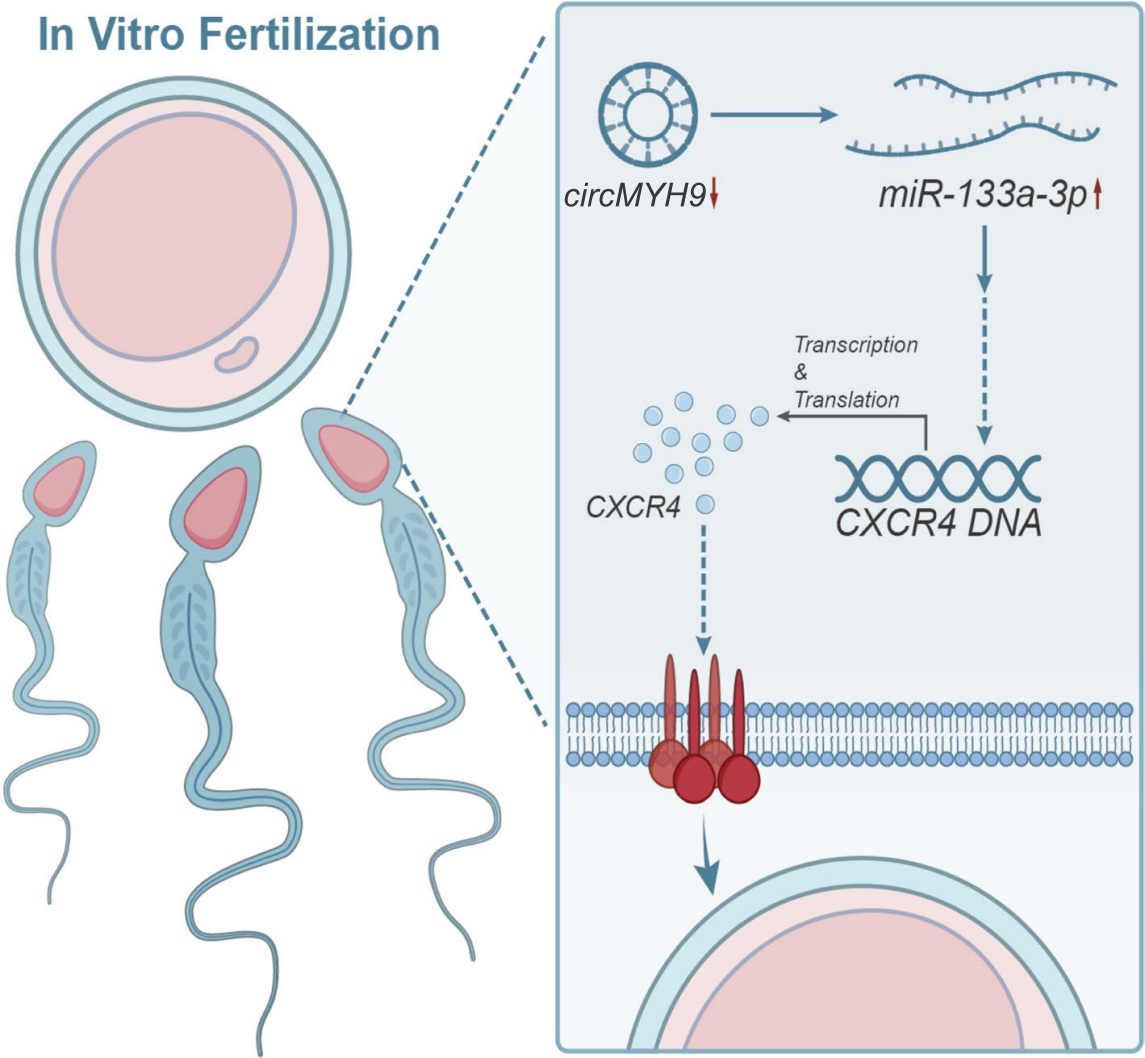
Molecular mechanism diagram of circMYH9/miR-133a-3p/CXCR4 regulatory network IVF of sperm

However, our study has several limitations. Firstly, this study primarily utilized the mouse spermatogonial cell line GC-2spd as the experimental model. Yet, differences exist between the reproductive systems of mice and humans, necessitating further validation of whether these findings can be directly applied to humans. Secondly, although this study identified the circMYH9/miR-133a-3p/CXCR4 regulatory network, it may only represent a fraction of the regulatory processes during reproduction. Hence, further research is required to unveil other potential regulatory molecules or pathways. While numerous in vitro and in vivo experiments have confirmed the function of miR-133a-3p, additional mechanistic studies and in-depth functional validations are still necessary. Future research could delve deeper into the circMYH9/miR-133a-3p/CXCR4 regulatory network to uncover more critical molecules and pathways. Expanding the scope of the research to include other animal models and humans could validate the universality and applicability of these findings. Exploring how to translate the discovery of this regulatory network into clinical applications to provide more effective diagnosis and treatment methods for infertility patients is crucial (Fig. 9).

## Materials and methods

### Sample collection

Patients treated at Xuzhou Central Hospital and Shuyang Hospital from August 2022 to May 2023 were included in this study. Couples with female infertility caused by tubal factors or unexplained reasons were divided into experimental and control groups. Females aged 20–44 were included, while males with abnormal semen parameters, smoking, alcohol abuse, chronic medication, infections, or tumors were excluded. Clinical data information was recorded (Table S1).

Semen was collected after 3 days of abstinence, processed through density gradient centrifugation, and stored for transcriptome sequencing. Patients with FR below 50% were placed in Group A, and those with FR above 50% in Group B. The study was approved by the Clinical Ethics Committee of Xuzhou Central Hospital (XZXY-LK-20230822–0147), and patients were informed and agreed to participate in adherence to the Helsinki Declaration guidelines [23].

### RNA sequencing

RNA was extracted from sperm samples using Trizol reagent (Thermo Fisher, 16096020). RNA quality was assessed via the Qubit®2.0 Fluorometer® (Life Technologies, Q33216), the Qubit® RNA assay kit (Shanghai Baoji Biotechnology, HKR2106-01), and the Bioanalyzer 2100 system (Agilent, 5067–1511) [24, 25]. For mRNA sequencing, the NEBNext® UltraTM RNA Library Prep Kit (NEB, E7435L) was used, and libraries were generated on the Illumina-HiSeq 2000 platform. Paired-end reads were processed using FastQC software, followed by adapter trimming with Cutadapt, and sequence filtering using FASTX Toolkit [24–26]. HISAT2 (v0.7.12) was used to align reads to the human genome. CircRNA sequencing involved enrichment and RNase A digestion, followed by cRNA generation and quantification via a circRNA microarray (Arraystar). For miRNA sequencing, the samples were processed with the SOLiD Small RNA Expression Kit (Applied Biosystems), followed by purification, adapter ligation, reverse transcription, and PCR amplification. The products were sequenced using the SOLiD 4 system and analyzed against miRNA databases [27, 28].

### Disease characteristic gene screening

The "edgeR" package in R software was used to filter circRNA, miRNA, and mRNA data to identify differentially expressed genes between the experimental (A) and control (B) groups. The criteria were |logFC|> 1 and *P*-value < 0.05. R's "heatmap" and "ggplot2" packages were used to visualize differential expression. Differential miRNAs based on available embryo rate (AER) were identified and intersected with those based on FR. The jvenn database was employed to generate Venn diagrams of shared disease-associated miRNAs. GO and KEGG enrichment analysis was performed on mRNAs using the "clusterProfiler" package, and protein–protein interaction (PPI) analysis was conducted using the STRING database to identify key regulatory genes [29, 30].

### Construction of competing endogenous RNA (ceRNA) network

A comprehensive ceRNA network was constructed using Starbase to predict mRNA interactions with disease-specific miRNAs. These mRNAs were intersected with core genes from PPI analysis. Additionally, miRNA-circRNA binding predictions were made, and key circRNAs were identified. The ceRNA network was built using Cytoscape software to illustrate the interactions between mRNA, miRNA, and circRNA [31, 32].

### Formation and nurturing of cellular models in vitro

The GC-2spd cell line (CRL-2196, ATCC) was cultured in DMEM medium containing high glucose (11965092, Gibco) and supplemented with 10% fetal bovine serum (FBS, 10099141C, Gibco) at 37°C with 5% CO_2_ in a humidified incubator (Heracell™ Vios 160i CR CO_2_ incubator, Thermo Scientific, catalogue number 51033770). Cell passaging was performed when the cells reached 80%-90% confluence. After three passages, the cells were assessed for optimal conditions. Once 80%-90% confluence was reached, the cells were washed with phosphate-buffered saline (PBS) and digested with trypsin. The digestion process was halted by adding a complete medium. After centrifugation, the supernatant was discarded, and 1 mL of freezing solution was added. The cell mixture was then transferred to cryogenic vials and stored at -80 °C or in liquid nitrogen for long-term preservation.

The 293 T cell line (CRL-3216, ATCC) was cultured in DMEM with 10% FBS, 10 μg/mL streptomycin, and 100 U/mL penicillin at 37 °C with 5% CO_2_. Cell passaging was performed when cells reached 80%-90% confluence [33–35].

### Construction of lentiviral vectors

Lentiviruses overexpressing circMYH9 and CXCR4 were assembled by Genechem Company (Shanghai), using the LV-PDGFRA vector. The miR-133a-3p overexpression and silencing lentiviruses were created with a mimic and inhibitor from Guangdong RayBiotech Co., Ltd. The packaged virus and target vector were transfected into 293 T cells using Lipofectamine 2000 when cells reached 80–90% confluence. After 48 h of incubation, the viral supernatant was collected, and viral titers were measured. Lentivirus was added to 293 T cells at a density of 5 × 10^4^ cells/mL in 6-well plates, with stable strains selected using 2 μg/mL puromycin (Sigma-Aldrich) over 14 days [36–39].

### Cell transfection

Cell culture was maintained until 80–90% confluence was reached. Cells were washed 2–3 times with PBS and digested with trypsin, after which the digestion was stopped by adding a complete culture medium containing 10% FBS. The cell suspension was transferred to a 15 mL centrifuge tube and centrifuged at 1000 rpm for 5 min. After centrifugation, the supernatant was removed, and 3 mL of complete culture medium was added to the cell sediment. Cells were thoroughly resuspended by gentle pipetting, followed by cell counting. Based on the results, the cell density was adjusted, and the cells were seeded evenly into 6-well or 12-well plates. Cells were placed in an incubator for cultivation. In RT-qPCR experiments, transfection efficiency was validated using 12-well plates, while 6-well plates were used for Western blot experiments.

The groups for transfection were as follows: (1) mimic-NC group (miRNA mimic negative control); (2) inhibitor-NC group (miRNA inhibitor negative control); (3) miR-133a-3p mimic group (transfected with miR-133a-3p mimic); (4) miR-133a-3p inhibitor group (transfected with miR-133a-3p inhibitor); (5) oe-NC + mimic-NC group (lentiviral empty vector + miRNA mimic negative control); (6) oe-MYH9 + mimic-NC group (oe-MYH9 plasmid + miRNA mimic negative control); (7) oe-MYH9 + mimic-miR-133a-3p group (oe-MYH9 plasmid + miR-133a-3p mimic); (8) mimic-miR-133a-3p + oe-NC group (miR-133a-3p mimic + empty vector); (9) mimic-miR-133a-3p + oe-CXCR4 group (miR-133a-3p mimic + oe-CXCR4 plasmid); (10) oe-NC + sh-NC group (overexpressed and silenced non-coding MYH9); (11) oe-circMYH9 + sh-NC group (oe-circMYH9 plasmid + silenced non-coding MYH9); (12) oe-circMYH9 + sh-CXCR4 group (oe-circMYH9 plasmid + sh-CXCR4 plasmid). The effectiveness of each transfection was verified after 48 h via RT-qPCR [27].

### RT-qPCR

RNA was extracted using Trizol reagent (Invitrogen), and its concentration and purity were assessed with NanoDrop LITE (Thermo Scientific). The PrimeScript RT reagent Kit (TaKaRa) was used to synthesize cDNA. Total RNA was also isolated using the miRNeasy micro kit (QIAGEN) for miR-133a-3p expression analysis, followed by cDNA synthesis with the miRNA First Strand cDNA Synthesis Kit (Shanghai Sangon Biotechnology). RT-qPCR detection used SYBR Green PCR Master Mix (Applied Biosystems) and the ABI PRISM 7500 system. Primers were synthesized by Shenggong Biotechnology based on Table S2. miR-133a-3p expression was normalized using U6, while other genes were normalized to GAPDH. The relative expression levels of DLEU2 and other genes were analyzed using the 2^−ΔΔCt^ method, calculated as ΔΔCt = (target gene Ct in the experimental group—housekeeping gene Ct in the experimental group)—(target gene Ct in the control group—housekeeping gene Ct in the control group). All RT-qPCR experiments were repeated thrice [40–43].

### Dual-luciferase reporter gene experiment

The binding sites for miR-133a-3p and circMYH9/CXCR4 were retrieved from bioinformatics databases. Wild-type (wt) and mutant (mut) sequences for the 3'-UTR regions of circMYH9 and CXCR4 were designed. These sequences were inserted into the pmiR-GLO-ReportTM vector (Biosage). The plasmids containing wt and mut sequences, along with miR-133a-3p mimic and mimic-NC plasmids, were transfected into 293 T cells using Lipofectamine 2000. After 48 h, the cells were lysed, and the supernatant was collected. The Dual-Luciferase Reporter Assay System (Promega) was used to analyze relative luciferase activity. The experiment was performed in triplicate for reliability [44].

### RNA pull-down

Cells transfected with Bio-miR-133a-3p-wt and Bio-miR-133a-3p-mut RNA (50 nM) were collected after 48 h. After washing with PBS, the cells were lysed in a buffer containing protease and RNase inhibitors. The lysate was incubated overnight at 4℃ with streptavidin-coated magnetic beads pre-coated with RNase-free BSA and yeast tRNA. After incubation, the expression levels of circMYH9 and CXCR4 were detected using RT-qPCR analysis. The experiment was repeated three times for accuracy [45].

### MTT assay

The test cells were inoculated onto a 96-well cell culture plate at a density of 3–5 × 10^4^ cells/mL, followed by a 48-h incubation period. Add MTT solution (10 mg/mL, Cat. No. ST316, Beyotime Biotechnology Co., Ltd, Shanghai) to the cell suspension, incubate for 4 h, then add dimethyl sulfoxide (DMSO) and mix for 10 min. The spectrophotometer from Laspec, China, was employed for measuring absorbance at 490 nm [46].

### EdU experiment

Cells were seeded in a 24-well plate, and EdU (10 µmol/L, Guangzhou RiboBio Co.) was added to the medium for a 2-h incubation in a CO_2_ incubator. After medium removal, cells were fixed with 4% formaldehyde for 30 min at room temperature. The cells were washed with PBS containing 3% BSA, followed by treatment with 0.5% Triton-100 for 20 min to enhance permeability. After washing, 100 µL of staining solution was added to each well, incubating in the dark for 30 min at room temperature. DAPI was added for nuclear staining and incubated for 5 min. Randomly selected stained cells were analyzed using a fluorescence microscope (IX73, Olympus), and the number of EdU-positive cells was assessed using Image-Pro Plus 6.0 software. The EdU labeling rate was calculated as the percentage of positive cells. The experiment was performed in triplicate [47].

### TUNEL staining

GC-2spd cells were fixed with 4% paraformaldehyde (Shanghai YESBIOTECH) for 15 min and permeabilized with 0.25% Triton X-100 for 20 min at room temperature. After blocking with 5% BSA (Shanghai Yisheng Biological Technology), the cells were stained with TUNEL reagent (Beyotime Biotechnology) and DAPI. Apoptotic cells were imaged using a confocal microscope (LSM 880, Carl Zeiss). TUNEL-positive cells emitted green fluorescence (apoptotic), while DAPI-stained nuclei appeared blue. The apoptosis rate was calculated by dividing the number of TUNEL-positive cells by the total number of cells, based on five different fields [48].

### Western blot

Cells were lysed with RIPA buffer (Beyotime) containing protease inhibitors for total protein extraction. Protein concentrations were determined using the BCA protein quantification kit (Beyotime). After denaturation, proteins were separated by 10% SDS-PAGE electrophoresis and transferred to a PVDF membrane. The membrane was blocked with 5% BSA for 2 h at room temperature to prevent nonspecific binding. Afterward, primary antibodies (details in Table S3) were incubated for 1 h, followed by washing and incubation with goat anti-rabbit secondary antibody (HRP-conjugated, Abcam). The membrane was exposed using Pierce™ ECL Western Blot Substrate (Thermo Scientific) and imaged using a Bio-Rad imaging system. Grayscale values of the Western blot bands were analyzed with ImageJ software, with GAPDH used as the internal reference [41]. Each experiment is repeated 3 times.

### In vivo animal experiments

Eight-week-old male C57BL/6 J mice (*n* = 18) were sourced from Beijing Vital River Laboratory Animal Technology Co., Ltd. (Catalog#: 219, Beijing, China) and placed in a specific-pathogen-free (SPF) animal facility under controlled conditions (humidity: 60%-65%, temperature: 22–25 °C) after a one-week acclimatization period. Observations on the health status of the mice were made prior to the commencement of the experiments. These experiments were performed with the necessary approval from the Institutional Animal Care and Use Committee. Anesthesia was initiated for the mice by intraperitoneal injection of copper fluoride (0.4 ml/kg, Sigma, Catalog#: SML1875) and diazepam (5 mg/kg, Catalog#: 439–14-5), followed by shaving and disinfection of the scrotal region. Subsequently, the scrotal skin was exposed, and the epididymides were carefully dissected and isolated. A precise volume of lentiviral vector (200 μL per side) was injected into both epididymides using a micropipette, followed by suturing and disinfection. After a 14-day period of postoperative care in a sterile animal facility, the mice were utilized for subsequent experiments [49].

The experimental groups were as follows: oe-NC + mimic-NC, oe-circMYH9 + mimic-NC, and oe-circMYH9 + mimic-miR-133a-3p, each consisting of 6 mice per group [49]. The expression levels of the corresponding proteins in mouse sperm and sperm viability were determined by qRT-PCR and IVF assays, respectively.

### Separating sperm

The epididymis and vas deferens were extracted from male mice and transferred into a culture dish. The dish was coated with liquid paraffin and contained 500 μL of GIVF Plus medium (Vitrolife AB, Sweden). The tissue was surgically cut, and the sperm were extracted and placed into the culture medium. The sperm were then incubated at 37 °C and used for IVF experiments following energy acquisition [50].

### Sperm motility analysis

Male mice were anesthetized with 1% sodium pentobarbital and euthanized by decapitation. The bilateral epididymides were removed and placed in pre-warmed human tubal fluid (HTF) at 37 °C. The caudal epididymis was cut, and mature sperm were released into the HTF. A 10 μL sample containing sperm was analyzed using a CASA microscope system to measure sperm motility, including progressive motility (PR), non-progressive motility (NP), immotile sperm, total motility rate (PR + NP), curvilinear velocity (VCL), straight-line velocity (VSL), average path velocity (VAP), and beat cross frequency (BCF) [51].

### IVF

To retrieve oocytes, 6-week-old female C57BL/6 J mice were injected with human chorionic gonadotropin (hCG) for superovulation and euthanized 15–18 h later. After exposing the uterus, fallopian tubes, and ovaries, the fallopian tubes were cleaned and placed in paraffin oil. The egg mass was gently expressed from the enlarged fallopian tube section into the oil. The dish containing the ovum clumps was incubated at 37 °C with 5% CO_2_ for 20–30 min. Capacitated sperm (1–2 μL) was added to the droplet containing the oocytes, and Fertilization occurred over 3 h. Post-incubation, the oocytes were washed three times with GIVF Plus medium. The IVF FR was calculated as the ratio of fertilized embryos to total eggs, while the 2-cell development rate was determined as the ratio of embryos developing into 2-cell stages to the total number of eggs [52].

### Delayed imaging analysis of embryo development

After IVF of mouse embryos obtained from IVF 4 h post-fertilization, the sperm attached to the zona pellucida was removed using a pipette. Fertilized eggs should exhibit at least one polar body as an indication of MII oocyte. Primovision (EmbryoScope monitoring system) was used to capture time-lapse images of the development of fertilized eggs prior to implantation. The fertilized eggs were cultured in G-TL™ medium (Vitrolife, https://www.vitrolife.com) for 96 to 100 h, with photographs taken every 10 min. Data collection was performed using Primovision Analyzer software [52].

### Statistical analysis

Expression data for miRNA, mRNA, and circRNA were obtained through high-throughput sequencing. Differentially expressed genes between the experimental and control groups were identified using the "edgeR" package in R software, with selection criteria of |logFC|> 1 and *P*-value < 0.05. The selected differentially expressed genes were further analyzed using GO and KEGG enrichment to reveal potential biological processes and signaling pathways associated with IVF and embryo development. Protein–protein interaction (PPI) analysis was performed using the STRING database to identify key regulatory genes. Additionally, disease-specific miRNA binding sites with mRNA and circRNA were predicted using the StarBase database, and a competing endogenous RNA (ceRNA) network was constructed using Cytoscape software to illustrate the interactions between mRNA, miRNA, and circRNA.

The experimental data were statistically analyzed using SPSS 26.0 software, and the generation of bar graphs was accomplished using Graphpad Prism software. All measurement data were represented as mean ± standard deviation. Before conducting between-group comparisons, data normality and homogeneity of variance were verified to ensure normal distribution and equal variances. Independent samples were analyzed using an independent t-test. In cases of multiple-group comparisons, a one-way analysis of variance (One-Way ANOVA) was used to determine intergroup differences, followed by Tukey’s post hoc test. Statistical significance was inferred at a *P*-value < 0.05.

## Supplementary Information

Supplementary Material 1: Figure S1. Bioinformatics analysis for selection of differentially expressed circRNA and mRNA during IVF of sperm. Note: (A) Volcano plot showing differentially expressed circRNA, with red indicating upregulated genes and blue indicating downregulated genes, *n*=2 for the control group, *n*=2 for the treated group; (B) Volcano plot showing differentially expressed mRNA, with red indicating upregulated genes and blue indicating downregulated genes, *n*=3 for control group, *n*=3 for treated group; (C) Heatmap showing differentially expressed circRNA, with red indicating upregulated genes and blue indicating downregulated genes, A represents the treated group, B represents the control group; (D) Heatmap showing differentially expressed mRNA, with red indicating upregulated genes and blue indicating downregulated genes, A represents the treated group, B represents the control group. Figure S2. PPI analysis of differentially expressed mRNA.Note: The figure shows key proteins in the PPI network of differentially expressed mRNA.

## Data Availability

The data supporting this study's findings are available from the corresponding author upon reasonable request. The materials presented are contained within the manuscript.
