# Supplementary material for: CircMYH9/miR-133a-3p/CXCR4 axis: a novel regulatory network in sperm fertilization and embryo development
Source: Mol Biomed. 2024 Dec 13;5:69. doi: 10.1186/s43556-024-00236-5 (PMC11645365; doi:10.1186/s43556-024-00236-5)
Supplement: Supplementary file 1 — Supplementary Material 1: Figure S1. Bioinformatics analysis for selection of differentially expressed circRNA and mRNA during IVF of sperm. Note: (A) Volcano plot showing differentially expressed circRNA, with red indicating upregulated genes and blue indicating downregulated genes, n=2 for the control group, n=2 for the treated group; (B) Volcano plot showing differentially expressed mRNA, with red indicating upregulated genes and blue indicating downregulated genes, n=3 for control group, n=3 for treated group; (C) Heatmap showing differentially expressed circRNA, with red indicating upregulated genes and blue indicating downregulated genes, A represents the treated group, B represents the control group; (D) Heatmap showing differentially expressed mRNA, with red indicating upregulated genes and blue indicating downregulated genes, A represents the treated group, B represents the control group. Figure S2. PPI analysis of differentially expressed mRNA.Note: The figure shows key proteins in the PPI network of differentially expressed mRNA. [file 43556_2024_236_MOESM1_ESM.docx]

**CircMYH9/miR-133a-3p/CXCR4 Axis: A Novel Regulatory Network in Sperm Fertilization and Embryo Development**

**Running Title:** miR-133a-3p inhibits sperm fertilization and embryo development in IVF

Qian Sun**^1,2,#^**, Yanyu Li**^1,3,4,#^**, Wen Yang**^2^**, Wen Feng**^2^**, Jiayun Zhou**^4^**, Yijuan Cao**^5^**, Bei Zhang**^1,3,4*^**, Zuobin Zhu**^10,*^**, Conghui Han**^3,6,7,8,9,*^**

**^1^**Suzhou Medical College, Soochow University, Suzhou 215123, China.

**^2^**Department of Gynecology, The First Affiliated Hospital of Kangda College of Nanjing Medical University, Lianyungang 222061, China.

**^3^**Clinical Medicine Postgraduate Workstation of Soochow University, Xuzhou 221009, China.

**^4^**Department of Gynecology, Xuzhou Central Hospital, Xuzhou 221009, China.

**^5^**Department of Reproductive Medicine, Xuzhou Central Hospital, Xuzhou 221009, China.

**^6^**Department of Urology, Xuzhou Central Hospital, Xuzhou 221009, China.

**^7^**Department of Urology, Xuzhou Clinical School of Xuzhou Medical University, Xuzhou 221009, China.

**^8^**School of Life Sciences, Jiangsu Normal University, Xuzhou 221116, China.

**^9^**Department of Urology, Heilongjiang Provincial Hospital, Harbin 150006, China.

**^10^**Xuzhou Engineering Research Center of Medical Genetics and Transformation, Key Laboratory of Genetic Foundation and Clinical Application, Department of Genetics, Xuzhou Medical University, Xuzhou 221004, China.

^#^ These authors are regarded as co-first authors

**^*^ Correspondence to:**

**Bei Zhang** (corresponding author)**,** Suzhou Medical College, Soochow University, Suzhou 215123, China; Clinical Medicine Postgraduate Workstation of Soochow University, Xuzhou 221009, China; Department of Gynecology, Xuzhou Central Hospital, [No. 199, South Jiefang Road](https://cn.bing.com/maps?&mepi=109~~TopOfPage~Address_Link&ty=18&q=%E5%BE%90%E5%B7%9E%E5%B8%82%E4%B8%AD%E5%BF%83%E5%8C%BB%E9%99%A2&ss=ypid.YN4067x10204286911338516522&ppois=34.240943908691406_117.19510650634766_%E5%BE%90%E5%B7%9E%E5%B8%82%E4%B8%AD%E5%BF%83%E5%8C%BB%E9%99%A2_YN4067x10204286911338516522~&cp=pq647dtrzmzp&v=2&sV=1&FORM=MPSRPL), Quanshan District, Xuzhou 221009, China.

**E-mail:** bettyzhang10@163.com

**Tel.:** +86-18952171130

**Zuobin Zhu** (corresponding author)**,** Xuzhou Engineering Research Center of Medical Genetics and Transformation, Key Laboratory of Genetic Foundation and Clinical Application, Department of Genetics, Xuzhou Medical University, Xuzhou 221004, China.

**E-mail**: zhuzuobin@xzhmu.edu.cn

**Tel.:** +86-18051369509

**Conghui Han** (corresponding author)**,** Clinical Medicine Postgraduate Workstation of Soochow University, Xuzhou 221009, China; Department of Urology, Xuzhou Clinical School of Xuzhou Medical University, Department of Urology, Xuzhou Central Hospital, Xuzhou 221009, China; School of Life Sciences, Jiangsu Normal University, Xuzhou 221116, China; Department of Urology, Heilongjiang Provincial Hospital, Harbin 150006, China.

**E-mail**: hanchdoctor@st.btbu.edu.cn

**Tel.:** +86-13813461893

**
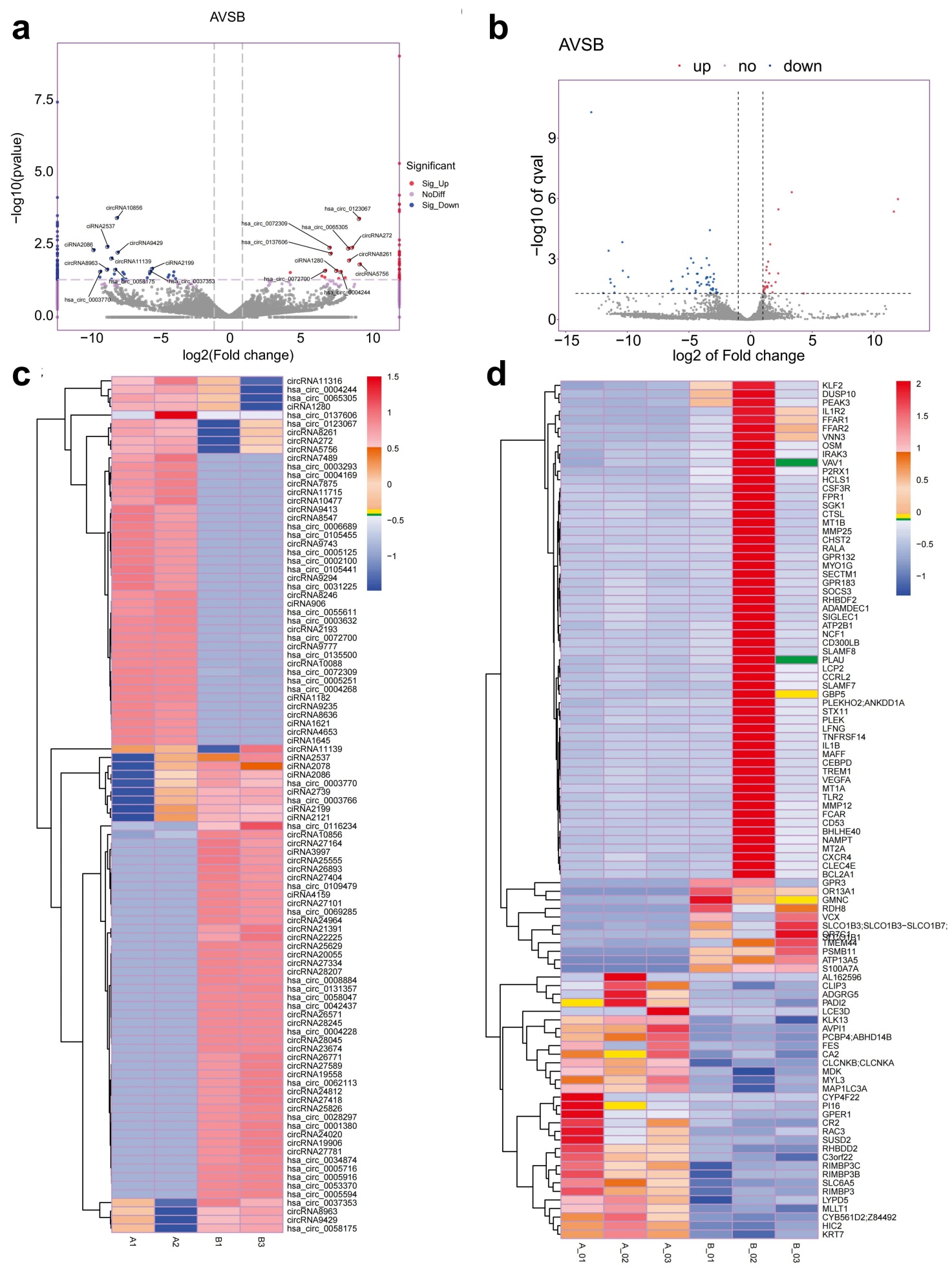
**

**Figure S1. Bioinformatics analysis for selection of differentially expressed circRNA and mRNA during IVF of sperm.**

Note: (A) Volcano plot showing differentially expressed circRNA, with red indicating upregulated genes and blue indicating downregulated genes, n=2 for the control group, n=2 for the treated group; (B) Volcano plot showing differentially expressed mRNA, with red indicating upregulated genes and blue indicating downregulated genes, n=3 for control group, n=3 for treated group; (C) Heatmap showing differentially expressed circRNA, with red indicating upregulated genes and blue indicating downregulated genes, A represents the treated group, B represents the control group; (D) Heatmap showing differentially expressed mRNA, with red indicating upregulated genes and blue indicating downregulated genes, A represents the treated group, B represents the control group.

**
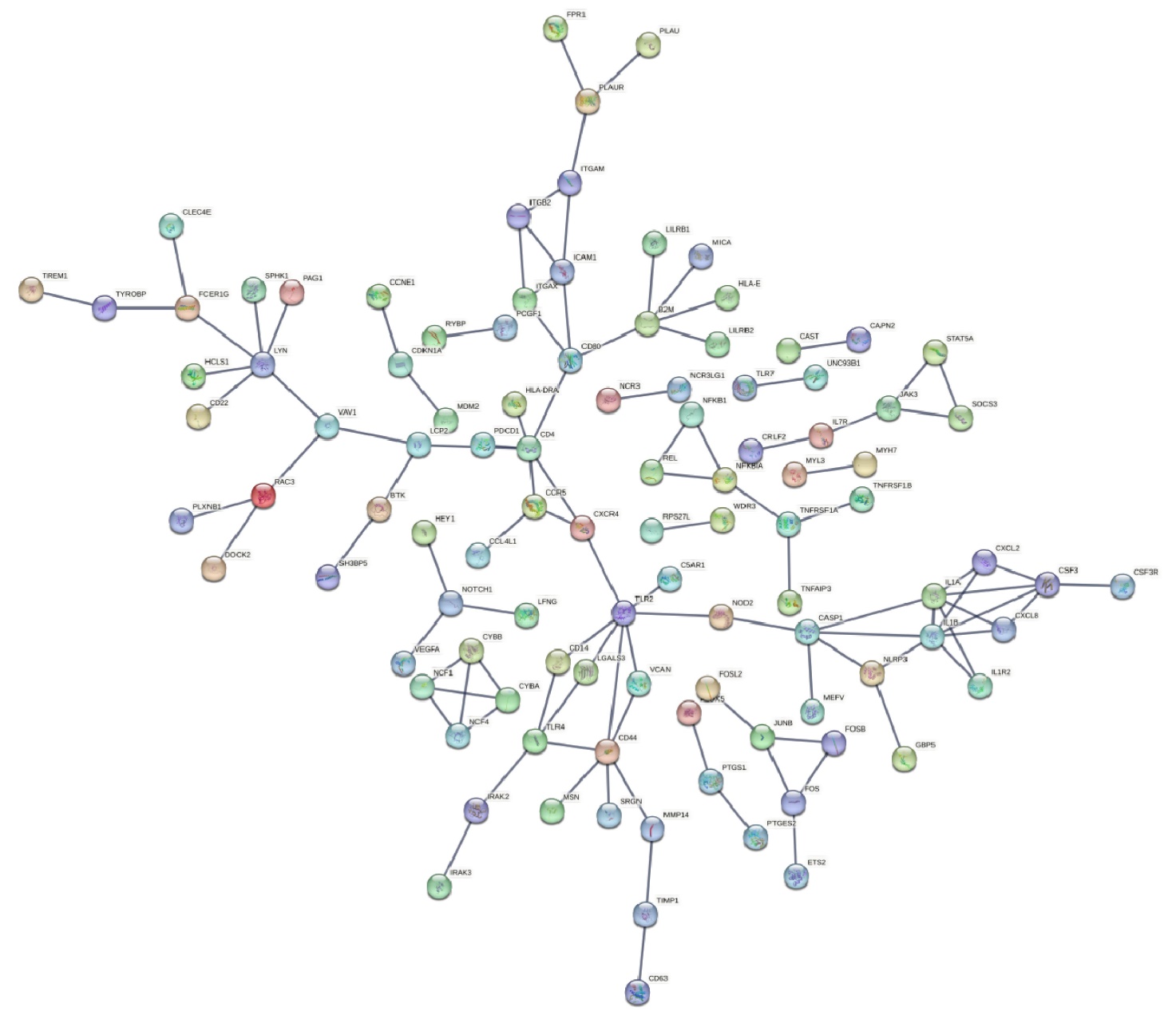
**

**Figure S2. PPI analysis of differentially expressed mRNA.**

Note: The figure shows key proteins in the PPI network of differentially expressed mRNA.

**Table S1. Clinical data**

| Serial Number | Male age | Fertility（FR） | Effective embryo rate（EER） |
| --- | --- | --- | --- |
| A_01 | 30 | 38.9% | 57.1% |
| A_02 | 26 | 20% | 100% |
| A_03 | 32 | 36.8% | 85.7% |
| B_01 | 33 | 100% | 57.1% |
| B_02 | 34 | 100% | 69.2% |
| B_03 | 33 | 100% | 100% |

Note: Grouping is based on fertilization rate, with experimental groups consisting of A_01, A_02, A_03 with fertilization rates below 50%; B_01, B_02, B_03 with a fertilization rate higher than 50% are the control groups.

**Table S2. RT-qPCR Primer sequence(mice)**

| Gene | Primer sequence |
| --- | --- |
| MYH9 | Forward: 5’- AGAAGTTGGTATGGGTGCCTT -3’ |
|  | Reverse: 5’- CCCTGAGTAGTATCGCTCCTTG -3’ |
| miR-133a-3p | Forward: 5’-TTTGGTCCCCTTCAACCAGCTG-3’ |
|  | Reverse: universal primer |
| CXCR4 | Forward: 5’-TGCAGCAGGTAGCAGTGAAA-3’ |
|  | Reverse: 5’-TCGGTTCCATGGCAACACTC-3’ |
| U6 | Forward: 5’-CTCGCTTCGGCAGCACATA-3’ |
|  | Reverse: 5’-AACGATTCACGAATTTGCGT-3’ |
| GAPDH | Forward: 5’-GAGTCAACGGATTTGGTCGT-3’ |
|  | Reverse: 5’-GACAAGCTTCCCGTTCTCAG-3’ |

**Table S3. First antibody information**

| Name | Cat. | Dilution ratio | Manufacturer | Country |
| --- | --- | --- | --- | --- |
| Caspase-3 | Ab184787 | 1：2000 | Abcam | UK |
| cleaved Caspase-3 | Ab214430 | 1：5000 | Abcam | UK |
| Bax | Ab32503 | 1：1000 | Abcam | UK |
| Bcl-2 | Ab182858 | 1：2000 | Abcam | UK |
| GAPDH | Ab9485 | 1：2500 | Abcam | UK |
